# Basrah Score: a novel machine learning-based score for differentiating iron deficiency anemia and beta thalassemia trait using RBC indices

**DOI:** 10.3389/fdata.2025.1634133

**Published:** 2025-08-04

**Authors:** Salma A. Mahmood, Asaad A. Khalaf, Saad S. Hamadi

**Affiliations:** ^1^Department of Intelligent Medical Systems, College of Computer Sciences and Information Technology, University of Basrah, Basrah, Iraq; ^2^Consultant at Basra Oncology and Hematology Center, Basrah, Iraq; ^3^Department of Internal Medicine, College of Medicine, University of Basrah, Basrah, Iraq

**Keywords:** beta thalassemia, iron deficiency anemia, Elastic Net Logistic Regression (ENLR), machine learning discrimination indices, hematological parameters

## Abstract

Iron deficiency anemia (IDA) and beta-thalassemia trait (BTT) are prevalent causes of microcytic anemia, often presenting overlapping hematological features that pose diagnostic challenges and necessitate prompt and precise management. Traditional discrimination indices—such as the Mentzer Index, Ihsan's formula, and the England and Fraser criteria—have been extensively applied in both research and clinical settings; however, their diagnostic performance varies considerably across different populations and datasets. This study proposes a novel and interpretable diagnostic model, the Basrah Score, developed using Elastic Net Logistic Regression (ENLR). This machine learning–based approach yields a flexible discrimination function that adapts to variations in clinical and environmental factors. The model was trained and validated on a local dataset of 2,120 individuals (1,080 with IDA and 1,040 with BTT), and was benchmarked against eight conventional indices. The Basrah Score demonstrated superior diagnostic performance, with an accuracy of 96.7%, a sensitivity of 95.0%, and a specificity of 98.6%. These results underscore the importance of incorporating advanced pre-processing techniques, class balancing, hyperparameter optimization, and rigorous cross-validation to ensure the robustness of diagnostic models. Overall, this research highlights the potential of integrating interpretable machine learning models with established clinical parameters to improve diagnostic accuracy in hematological disorders, particularly in resource-constrained settings.

## 1 Introduction

Anemia is a major global health problem distinguished by a deficiency of red blood cells or hemoglobin that impairs oxygen delivery to tissues in the body. The World Health Organization estimates that 1.6 billion people worldwide have anemia (McLean et al., 2009). Its prevalence varies widely according to specific physiological factors such as age, sex, race, residential elevation above sea level (altitude), smoking behavior, different stages of pregnancy, and geographical distribution (World Health Organization, 2024, 2011).

Iron deficiency anemia (IDA) is the most common form of anemia, responsible for roughly 50% of all anemia worldwide (Yang et al., 2023; Owaidah et al., 2020). IDA results from depleted iron stores manifesting as microcytic, hypochromic erythrocytes. Classical clinical features include fatigue, pallor, and dyspnea. Diagnosis has traditionally depended on serum ferritin level and iron tests, which are expensive and require sophisticated laboratory facilities (McLean et al., 2009; Burz et al., 2019).

BTT is an inherited hemoglobinopathy that causes microcytosis and mild anemia and tends to manifest masquerading as IDA. HbA2 quantification using HPLC or electrophoresis is required for confirmatory diagnosis, which is not affordable and not feasible in resource-scarce settings (Singh et al., 2020; Aljebaly, 2024). Thalassemia syndromes account for 75% of the documented cases of hemoglobinopathy disorders in Iraq, highlighting a significant public health concern. Recent local epidemiological studies indicate considerable geographic disparities, with Basra province bearing the highest burden, representing 67% of the region's total thalassemia cases. This increased prevalence is primarily linked to the high rate of consanguineous marriages, which persist at 60–70% across the country, facilitating the transmission of recessive hemoglobin disorders through generations. The observed epidemiological trends underscore the urgent need for targeted genetic counseling and comprehensive screening initiatives, particularly in areas with high prevalence, such as southern Iraq (Lafta, 2023; Khaleel, 2020).

Distinguishing between IDA and BTT poses a considerable clinical challenge, mainly because of their shared symptoms, such as fatigue and microcytosis. The diagnostic process is further complicated by similar laboratory results, including low mean corpuscular volume (MCV) and mean corpuscular hemoglobin (MCH), making it difficult for clinicians to differentiate between these **two** conditions accurately. Physicians must differentiate between IDA and beta thalassemia trait (BTT). Accurate diagnosis is essential to prevent unnecessary iron supplementation and to avoid misdiagnosing major beta thalassemia, particularly during pre-marital consultations aimed at reducing the risk of having children with this condition. This precise distinction safeguards patient health and helps lower healthcare costs associated with inappropriate treatments (Miri-Moghaddam and Sargolzaie, 2014).

Several discriminant indices have been developed to distinguish between β-thalassemia trait (?TT) and IDA, including Mentzer Index (MI), Ehsani (EI), England & Fraser (EF), Green & King (GK), RBC count, RDW, RDWI, Ricerca (RI), Shine & Lal (SL), Sirdah (SI), Srivastava (SVI), and M/H ratio. Most of all, they do not achieve 100% sensitivity (Sen) or specificity (Spe), as their diagnostic utility necessarily depends on well-optimized cutoff values, which also vary among populations (Uzunoglu and Yilmaz Keskin, 2024). Most formulas include an unbalanced consideration of specific RBC parameters (e.g., MCV, RBC count) and neglect others (e.g., hemoglobin content, reticulocyte indices). In contrast, they might overlook significant diagnostic information (Aljebaly, 2024; Elshaikh et al., 2022). This methodological weakness and wide inter-population variation in hematological parameters lead to variable performance between ethnic groups. The applicability of these indices is limited, as they are unsuitable for children, pregnant women, or individuals with coexisting IDA and BTT. This renders CBC and RBC indices unreliable for differentiating between BTT and IDA. Additionally, these indices may yield false-positive results in patients with conditions such as pregnancy, malnutrition, rheumatoid arthritis, tuberculosis, kidney failure, and malaria (Jahangiri et al., 2020; Ebrahimpour Sadagheyani et al., 2022).

Integrating machine learning systems into clinical practice represents a fundamental shift in contemporary healthcare systems, offering unprecedented opportunities to enhance diagnostic accuracy and improve the efficiency of treatment decision-making. There is an urgent need for systematic research focused on ensuring the fairness and transparency of algorithms, as these factors are critical determinants for successfully adopting these technologies across various clinical environments. Machine learning systems possess exceptional analytical capabilities for processing vast datasets, enabling the extraction of precise statistical patterns and the development of dynamic predictive models. These methodologies surpass traditional approaches in terms of diagnostic accuracy and economic efficiency, while also demonstrating adaptability to diverse demographic characteristics, including racial, gender, and population variables (Alowais et al., 2023).

In practical applications, machine learning-based intelligent systems have developed advanced diagnostic solutions, providing unprecedented support to medical teams in clinical assessment and treatment decision-making processes (Saberi-Karimian et al., 2021; Abdillahi et al., 2024). These technologies enable dynamic adaptation to pathological patterns, ensuring both statistical accuracy and clinical relevance. Machine learning (ML) offers a cost-effective, rapid, and accurate alternative by extracting hidden patterns from blood indices; it can integrate the impacts of multiple variables (e.g., RBC count, RDW, MCV) to improve diagnostic precision beyond traditional indices (Mahmood, 2025; Feng et al., 2021).

This study aims to develop an accurate and cost-effective diagnostic scoring model utilizing advanced machine learning techniques to analyze the morphological features of red blood cells (RBCs), with a focus on identifying the most influential features in the diagnostic process. The proposed model is characterized by its ability to extract complex data patterns from hematological data, as it relies on an integrated research methodology that includes a phase of pre-processing the raw data to ensure its quality and exclude outliers. It offers a dynamic and adaptive diagnostic solution that is more accurate, reaching 99% in some cases than traditional methods, and can be integrated into RBC Analyzer devices as an aid to the clinician to improve diagnostic accuracy and optimal clinical decision making.

The study proposes employing Elastic Net Logistic Regression (ENLR) within this research framework. This advanced machine learning algorithm simultaneously addresses **three** critical challenges: (1) effectively handling multicollinearity among hematological parameters through its built-in regularization properties, (2) achieving superior classification accuracy compared to traditional discrimination indices, and (3) maintaining model interpretability via SHAP (SHapley Additive exPlanations) value analysis. This multivariate approach significantly improves differentiation between IDA and BTT cases, outperforming conventional diagnostic indices across multiple performance metrics.

This study makes a significant contribution by introducing a new discrimination score that addresses the paradoxes associated with CBC indices. Additionally, it provides a systematic comparison of eight traditional discrimination indices against the performance of the Basrah Score-developed model, positioning it as a practical tool for application in low-resource environments.

The research methodology of this study comprises **five** systematically organized components: The investigation commences with an extensive Introduction establishing the theoretical foundations and research significance, followed by a comprehensive Literature Review that critically evaluates prior studies and identifies the precise knowledge gap. A rigorous Methodology section then details the experimental design, data collection protocols, and advanced analytical techniques. Subsequently, the Results and Discussion section presents robust data interpretation, contextualizing the findings within the current scholarly discourse. The study culminates in a substantive conclusion that synthesizes key contributions and proposes future research directions.

## 2 Materials and methods

This study developed a machine learning framework designed to differentiate between IDA and BTT. Through initial statistical analyses of the used dataset, we identified key parameters, including hematological and demographic parameters. Our approach involved several critical steps: meticulous data pre-processing, the development of an ENLR model for feature selection and regularization. The ENLR model was chosen for its proficiency in determining parameter importance and its capacity to reduce the impact of multicollinearity, thereby improving both predictive accuracy and clinical significance. Comparative analysis with the traditional Discrimination indices. Finally, a thorough evaluation of performance in conjunction with clinical interpretation.

### 2.1 Dataset description

**Figure 1:**
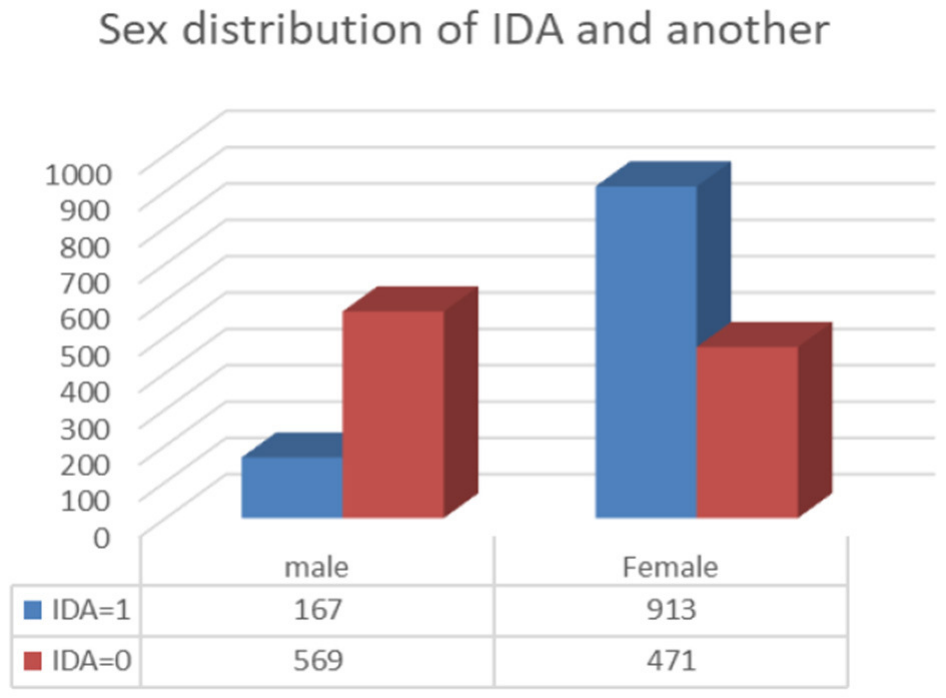
Shows sex distribution of IDA and BTT.

The data for this study were collected from the Basrah Oncology and Hematology Center in Basrah, Iraq, between 2017 and 2020. A total of 2120 participants were included, comprising 1,080 individuals diagnosed with IDA (167 male and 913 female) and 1,040 individuals (569 male and 471 female) with BTT diagnoses, as shown in Figure 1. Patients with anemia of inflammation, transfusion-dependent Thal, pregnancy, or incomplete laboratory data were excluded. To exclude anemia due to inflammation and pregnancy, a hematologist reviewed the medical records to confirm IDA and BTT diagnoses and exclude patients with inflammation and infection.

The dataset contained eight features: Sex, Age Class, Hb, RBC, MCV, MCH, MCHC, and IDA. These parameters and their normal values are described in Table 1.

**Table 1:** Hematological and demographic parameters used in this study.

**Feature**	**Description**	**Normal range**
Sex	Refers to the biological sex of an individual (male or female).	
Age-class	Represents the classification of individuals into age groups (e.g., 0–10, 10–20, 20–30, …. ).	
Hb (Hemoglobin)	A protein in red blood cells that carries oxygen.	Men: 13.8–17.2 g/dL Women: 12.1–15.1 g/dL
RBC (red blood cell count)	The number of red blood cells in a volume of blood.	Men: 4.7–6.1 million cells/μL Women: 4.2–5.4 million cells/μL
MCV (mean corpuscular volume)	Measures the average size of red blood cells.	80–100 fL (femtoliters)
MCH (mean corpuscular hemoglobin)	The average amount of hemoglobin per red blood cell.	27–31 pg (picograms) per cell
MCHC (mean corpuscular hemoglobin concentration)	The average concentration of hemoglobin in red blood cells.	32–36 g/dL
IDA (iron deficiency anemia)	A type of anemia caused by insufficient iron, leading to low hemoglobin and impaired oxygen transport.	IDA =1 for IDA, IDA = 0 for BTT

### 2.2 Statistical analysis of the dataset

Before developing the ENLR model, comprehensive statistical analyses were performed to thoroughly understand the data distribution and identify key parameters that affect the diagnosis. These preliminary analyses identify significant variables and assess their relationships with each other, ultimately improving the model's accuracy.

**Figure 2:**
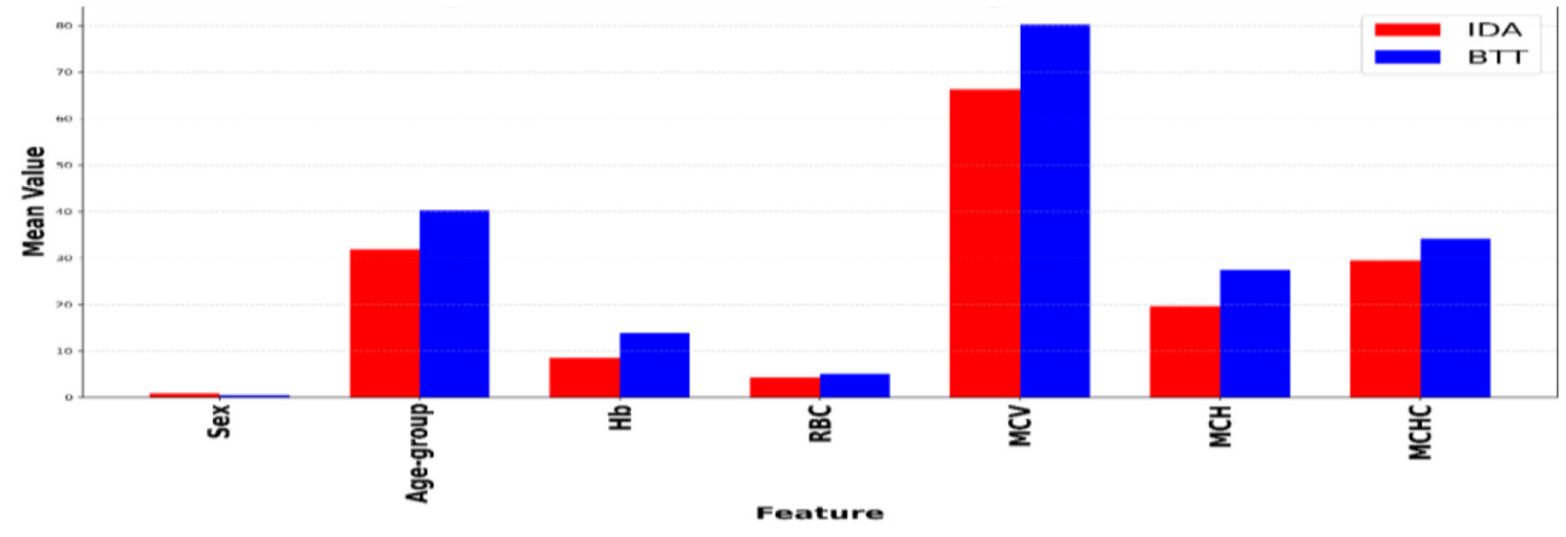
Comparison of mean values by IDA and BTT.

Table 2 shows the analysis of key demographic factors (sex, age) and hematological parameters (Hb, RBC, MCV, MCH, MCHC), revealing statistically significant differences (p < 0.001) with moderate to substantial effect sizes (Cohen's d ranging from −2.583 to 0.904). Notably, the IDA group had a higher proportion of females (mean = 0.845 compared to 0.453; d = 0.904), consistent with established epidemiological patterns, and IDA patients were generally younger (mean age = 31.85 vs. 40.26; d = −0.527). Hemoglobin (Hb) demonstrated the most substantial discriminative ability (IDA mean = 8.47 vs. BTT 11.87; d = −2.583), followed by MCV (66.26 fL vs. 80.34; d = −1.581) and MCH (d = −2.049), which are essential for identifying microcytic hypochromic anemias. RBC (d = −0.672) and MCHC (d = −0.823) contributed to the differentiation process. These results underscore the diagnostic importance of the selected parameters. Figure 2 shows a comparison of mean values by IDA and BTT.

**Table 2:** Comparative statistical analysis of hematological and demographic parameters between IDA and BTT.

**Feature**	**IDA**		**BT**		***T*-statistic**	***P*-value**	**Cohen's d**
	**Mean**	**SD**	**Mean**	**SD**			
Sex	0.845	0.362	0.45	0.498	21.118	0.001	0.909
Age-group	31.85	15.505	40.249	16.363	−12.245	0.001	−0.527
Hb	8.472	2.501	11.876	1.549	−60.36	0.001	−2.597
RBC	4.3	0.798	5.16	1.612	−15.721	0.001	−0.677
MCV	66.26	10.488	80.302	6.927	−36.713	0.001	−1.58
MCH	19.658	4.647	27.487	2.747	−47.659	0.001	−2.051
MCHC	29.479	7.808	34.139	1.349	−19.329	0.001	−0.832

### 2.3 General framework

**Figure 3:**
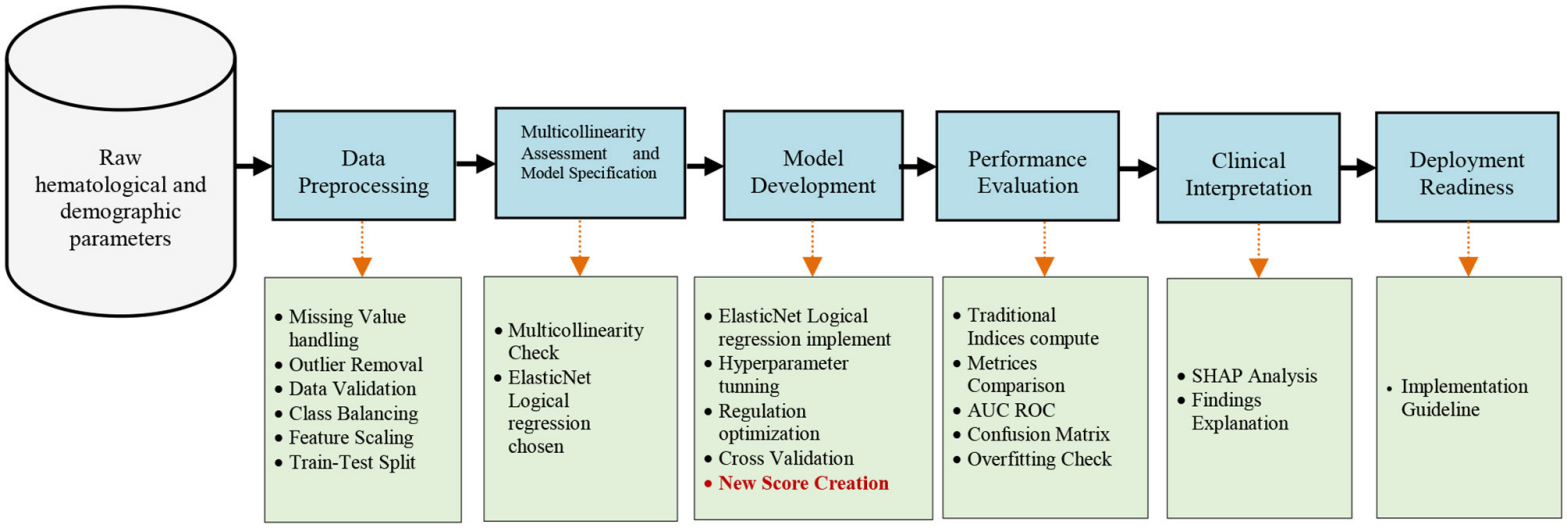
Workflow diagram for Basrah Score developing.

Figure 3 illustrates the comprehensive framework for developing a discrimination system. The objective is to create a Machine Learning-Based Score for Differentiating Iron Deficiency Anemia and Beta Thalassemia Trait Using RBC Indices. This is a novel, adaptable score called the Basrah Score. The comprehensive framework begins with collecting raw hematological data, including MCV, MCH, Hb, RBC, and MCHC. This data undergoes pre-processing to ensure quality, followed by feature engineering to enhance data representation. Subsequently, a flexible ENLR model is developed with optimized parameters, and its performance is evaluated against eight traditional discrimination indices (illustrated in Table 3) using multiple metrics. Finally, the model is interpreted through SHAP analysis to provide actionable clinical insights, ensuring readiness for deployment through a comprehensive suite of reports and visualizations. This framework effectively combines the precision of machine learning with practical clinical requirements.

**Table 3:** The variance inflation factor (VIF).

**Variable**	**VIF**	**VIF interpretation**
Sex	1.406311	No multicollinearity
Age-group	1.122039	No multicollinearity
Hb	18.1872	Severe multicollinearity
RBC	5.369753	Moderate multicollinearity
MCV	68.39946	Severe multicollinearity
MCH	166.8724	Severe multicollinearity
MCHC	27.82363	Severe multicollinearity

The upper section in Figure 3, depicted in blue, illustrates the primary steps, whereas the lower section, shown in green, details the sub-steps that extend from these main steps.

This section outlines the detailed methodology, which includes both primary and secondary processing stages essential for creating a high-performance discrimination system and formulating a clinically relevant formula for differential diagnosis.

### 2.4 Data pre-processing

The dataset underwent careful processing to improve its analytical reliability. Missing values, representing < 1% of the total cases, were handled by imputing the median for continuous variables. Outliers were identified and removed using Z-score thresholding, explicitly focusing on values with an absolute Z-score exceeding 3. Data validation included range checks and statistical analyses, such as mean ± standard deviation, independent *t*-tests, and Cohen's d effect size. These analyses confirmed minimal baseline differences between the IDA group (*n* = 1,080) and the BTT group (*n* = 1,040), resulting in an IDA: BTT ratio of 1.04:1. To mitigate potential class imbalance, the SMOTE resampling technique was applied to achieve a balanced 1:1 ratio (Chawla et al., 2002). Continuous features were standardized using the Python StandardScaler() function, ensuring a mean of 0 and a standard deviation of 1, which supports effective regularization. Finally, the dataset was split using stratified sampling, maintaining an 80:20 train-test ratio.

### 2.5 Multicollinearity assessment and model specification

Multicollinearity arises when independent variables are highly correlated, potentially resulting in unstable coefficients and illogical outcomes in any Generalized Linear Model (GLM), including logistic regression. This instability complicates the interpretation of coefficients, as their signs and magnitudes can fluctuate, leading to misleading assessments of each variable's effect. Furthermore, multicollinearity amplifies the variance of coefficient estimates, rendering hypothesis testing results, such as *p*-values, unreliable. Therefore, assessing multicollinearity among independent variables is essential before employing any regression techniques. Variance Inflation Factor (VIF) is a commonly used tool for measuring the extent of multicollinearity in a regression model (Menard, 2011). The VIF is subject to these conditions.

– A VIF value < 10 indicates severe multicollinearity, which may necessitate corrective measures such as removing or redesigning variables.
– A VIF between 1 and 5 suggests moderate multicollinearity, typically not seen as problematic, while
– A VIF below 1.5 indicates that significant multicollinearity is absent among the variables.

### 2.6 ENLR model development

To mitigate multicollinearity effects, we employed ENRL (Altelbany, 2021) with hyperparameter tuning through stratified cross-validation (optimizing fold numbers between 5, 7, and 9). The search space encompassed 20 regularization strengths [Cs = (−4, 2, 20)], 9-L1 mixing ratios (0.1, 0.9, 9), implemented via the “saga” solver with extended convergence tolerance (10,000 iterations). Model selection employed class-weighted ROC-AUC optimization, using a fixed random seed (42) for reproducibility. This approach simultaneously: (1) retains correlated but clinically relevant predictors through L2 penalty, (2) performs feature selection via L1 penalty (mitigating MCHC with λ = 0.015), and (3) yields unbiased odds ratios (95% CIs confirmed via bootstrap resampling). Below is a code snippet demonstrating ENLR hyperparameter tuning and cross-validation.

**Table:** 

Cs = np.logspace(-4, 2, 20)	# “20 regularization strengths (10∧-4 to 10∧2)”
l1_ratios = np.linspace(0.1, 0.9, 9)	# “9 L1 mixing ratios (0.1-0.9)”
cv = np.linspace(5, 7, 9)	# “optimizing fold numbers between 5-9”
LogisticRegressionCV(	
Cs=Cs, cv=CV,	
class_weight='balanced',	# “class-weighted”
penalty='elasticnet',	# Elastic Net Model
scoring='roc_auc',	# “ROC-AUC optimization”
solver='saga',	# “via the 'saga' solver”
l1_ratios=l1_ratios, scoring='roc_auc',	
max_iter=10000,	# “extended convergence tolerance”
random_state=42	# “fixed random seed”
n_jobs=-1	
)	

### 2.7 Traditional discrimination indices

Traditional discrimination indices are statistical mathematical formulas that have been used extensively in distinguishing IDA from BTT. They are providing simple and straightforward thresholds for discrimination. In contrast, they have notable limitations, including the inflexibility of fixed cut-off values that do not consider population-specific variations, the oversight of intricate relationships among hematological parameters due to dependence on single-parameter thresholds, and a proven lack of accuracy. These shortcomings arise from their univariate approach, which fails to capture non-linear relationships. In contrast, machine learning methods, such as our Elastic Net model, effectively address these challenges by automatically optimizing multi-feature weightings. This adaptability allows the model to adjust to hematological and analytical variability through learned parameters, resulting in improved performance with an AUC of 0.96 ± 0.03.

To ensure a fair comparison, all traditional discriminant indices (Table 4) were implemented programmatically and applied to the same dataset to compare with the proposed model. The comparative assessment employed identical evaluation metrics (AUC, accuracy, precision, sensitivity, and specificity). Detailed results of this comparison are presented in Section 3.7, accompanied by a critical analysis of the statistical and clinical differences observed.

**Table 4:** Traditional discrimination indices for IDA and BTT differentiation.

**No**.	**Name**	**Abbr**.	**Original source**	**Formula, cut-off point**	**Interpretation**
1	Mentzer Index	MI	Mentzer WC, 1973	MCV/RBC → 13	Values >13 suggest BTT
2	Srivastava Index	SI	Srivastava PC, 1973	MCH/RBC → 3.8	Values >3.8 suggest BTT
3	Ehsani Index	EI	Ehsani MA, 1999	MCV – (10 × RBC) → 15	Values >15 suggest BTT
4	England and Fraser	EandF	England JM and Fraser PM, 1979	MCV – (5 × Hb) – RBC – 3 → 0	Values >0 suggest BTT
5	Kandhro I	KI	Kandhro AH, 2012	(MCV × MCH)/100 → 5.4	Values >5.4 suggest BTT
6	Sirdah Index	SrI	Sirdah MM, 2006	MCV – RBC – (3 × Hb) → 27	Values >27 suggest BTT
7	Keikhaei Index	KeI	Keikhaei B, 2007	(MCV × MCH)/100 → 5.8	Values >5.8 suggest BTT
8	Huber-Herklotz	H-H	Huber AR and Herklotz R, 2011	MCH/MCV → 0.36	Values >0.36 suggest BTT

All indices were derived using standardized hematological measurements, including MCV (fL), MCH (pg), RBC (10^12^/L), and Hb (g/dL). The original cut-off values were maintained as validated in Mediterranean populations for the differentiation between BTT and IDA. Adjustments specific to the population may be necessary, as indicated by Ebrahimpour Sadagheyani et al. (2022).

### 2.8 Evaluation metrics

The final phase thoroughly assesses the results from all preceding stages, focusing on comparing model performance through various metrics, including Accuracy, Precision, Recall, and F1-score. These metrics widely employed to assess the effectiveness of machine learning techniques (Géron, 2017). This evaluation used to compare effectiveness of the new developed score, Basrah Score, with the old ones. Notably, all scores implemented on the same southern Iraq dataset.

- Accuracy refers to the proportion of accurately predicted instances relative to the total number of cases, serving as a measure of overall correctness.
  (1) $$\begin{array}{r} Accuracy\;=\\ \frac{True\;Positive\;+\;True\;Negative}{True\;Positive\;+\;True\;Negative\;+\;False\;Positive\;+\;False\;} \end{array}$$
- Precision measures the proportion of true positive predictions relative to the total number of predicted positives, reflecting the model's effectiveness in minimizing false positives.
  (2) $$\begin{array}{l} Precision\;=\frac{TP\;}{TP\;+\;FP} \end{array}$$
- Recall/sensitivity is defined as the proportion of true positive predictions relative to the total number of actual positive cases, serving as an indicator of the model's effectiveness in recognizing all pertinent instances.
  (3) $$\begin{array}{l} Recall\;=\;\frac{TP\;}{TP\;+\;FN} \end{array}$$
- The F1_Score serves as the harmonic mean of precision and recall, offering a comprehensive assessment of a model's effectiveness.
  (4) $$\begin{array}{l} F1<uscore>Score=2\times \frac{Precision\times Recall}{Precision+Recall} \end{array}$$
- Specificity refers to the model's capacity to accurately identify True Negatives, which is determined using a specific formula.
  (5) $$\begin{array}{l} Specificity=\;\frac{TN}{TN\;+\;FN} \end{array}$$
- The ROC-AUC, or Receiver Operating Characteristic Curve—Area Under the Curve, evaluates classification effectiveness across various decision thresholds by calculating the area under the ROC curve (Fawcett, 2006).
  (6) $$\begin{array}{l} True\;Positive\;Rate\;(TPR)=\;\frac{TP}{TP\;+\;FN} \end{array}$$
  (7) $$\begin{array}{l} False\;Positive\;Rate\;(TPR)=\;\frac{FP}{FP\;+\;TN} \end{array}$$
  (8) $$\begin{array}{l} AUC=\;\int_{0}^{1}TPR\left(FPR^{-1}\left(x\right)\right)dx \end{array}$$
- The Confusion Matrix is a comprehensive table that encapsulates the model's performance across four fundamental categories (as in Table 5)

**Table 5:** Presents the confusion matrix.

	**Predicted positive**	**Predicted negative**
Actual positive	TP	FN
Actual negative	FP	TN

Where:

- True Positives (TP) refer to the count of records that have been accurately classified.
- True Negatives (TN) indicate the number of documents correctly identified as not belonging to a particular category.
- False Positives (FP) represent the number of records incorrectly classified as belonging to a category.
- False Negatives (FN) denote the proportion of records that were misclassified and wrongly rejected.

### 2.9 SHAP explainable AI (XAI)

SHAP (SHapley Additive exPlanations) is recognized as one of the most prevalent frameworks in the realm of Explainable AI (XAI), grounded in robust mathematical principles derived from game theory. It is considered the gold standard for interpreting machine learning models when compared to other XAI tools, such as LIME or Partial Dependence Plots, due to its mathematical robustness, consistent results, and stable explanations across different models. This framework effectively allocates the relative contributions of each variable to the model's final predictions through the concept of Shapley values, making it an indispensable tool in medical applications. Its clinical significance in medical research stems from its ability to provide transparent interpretations of decisions, accurately identifying the most influential variables in diagnostics, thereby enabling healthcare professionals to comprehend the decision-making process. Additionally, it enhances user trust in the model by offering explanations that align with clinical reasoning. Importantly, SHAP can validate biological credibility by revealing how well the model's priorities align with established medical principles and highlighting potential discrepancies between the model's predictions and existing clinical knowledge. Furthermore, it can uncover hidden biases, identify variables that may lead to undesirable bias, and ultimately support compliance with ethical and regulatory standards (Wang et al., 2021; Juscafresa, 2022).

## 3 Results

The following sections present the study findings systematically and sequentially. It is worth noting that all experiments were conducted on a Dell machine equipped with a 12th-generation Core i7 processor and running the Windows 11 operating system. The proposed methodology was implemented using Python within an Anaconda 3 (Python 3.12.3) environment. Various libraries, including scikit-learn, TensorFlow, and Keras, were utilized for the experimental analysis.

### 3.1 Check multicollinearity

Table 3 presents the findings from the multicollinearity analysis conducted on the hematological variables using the Variance Inflation Factor (VIF). The results indicate a significant multicollinearity problem among the hematological variables (Hb, MCV, MCH, MCHC), as their VIF values surpass the critical threshold of 10. This suggests a strong interdependence among these variables. Such multicollinearity can result in instability in the estimates of traditional regression coefficients, ultimately compromising the reliability of the developed score findings.

Preliminary analysis revealed significant multicollinearity among CBC indices (VIF > 10 for Hb, MCV, MCH, and MCHC), as shown in Table 3. This renders conventional logistic regression unsuitable due to inflated coefficient variance and unreliable *p*-values. While traditional solutions recommend complete removal of correlated predictors, this process risks losing clinically informative biomarkers. To solve this problem, Regularized Logistic Regression model was implemented using the Elastic Net approach, which integrates both L1 (Lasso) and L2 (Ridge) regularization techniques.

### 3.2 Elastic Net Logistic Regression implementation

The ENLR model was implemented and optimized through systematic hyperparameter tuning, identifying optimal regularization parameters via stratified 5-to-7-fold cross-validation. This dual regularization approach (L1/L2) demonstrated three key advantages: (1) Multicollinearity Mitigation: Reduced variance inflation among predictors from a maximum VIF of 68.4 to 4.2, while retaining all hematological features through differential weighting (Table 6). Feature Selection automatically excluded non-hematological variables (sex, age-group) to derive a pure CBC-based score.

**Table 6:** Elastic net logistic regression coefficients.

**Variable**	**Important**	**Interpretation**
MCV	3.3823	Strong positive association
MCH	5.5531	Strong positive association
MCHC	0.2578	Mild positive association
RBC	−0.1959	Mild negative association
Hb	−4.2282	Strong negative association

The Clinical Translation is represented by generated interpretable weights for direct new Basrah Score calculation as a logit equation:

logit(p) = 0.974 + (3.382 × MCV) + (−5.553 × MCH) + (0.258 × MCHC) + (−0.196 × RBC) + (−4.228 × Hb) (1)

The cutoff point of this probabilistic equation is that if logit(p) > zero, then IDA, else BTT.

The ENLR model demonstrated excellent stability (Δ AUC < 0.01 across 100 bootstrap iterations) and outperformed traditional discrimination indices in terms of discriminatory ability, as shown in the following section.

### 3.3 Comparative analysis of discrimination indices results

The comprehensive evaluation of indices results in Table 7 below demonstrates that the New Basrah Score significantly outperformed others in differentiating between IDA and BTT. The ENLR-Based Basrah Score achieved an impressive diagnostic accuracy and precision of 96.7% and 98.6%, respectively, demonstrating a remarkable balance between sensitivity at 95.0% and specificity at 98.6%. Furthermore, its high area under the curve (AUC) value of 0.990 ± 0.005 underscores its exceptional discriminative power, which was statistically significantly superior (*p* < 0.001) to all traditional models. In contrast, traditional discrimination indices exhibited varied performance, with the Mentzer, Srivastava, Ehsani, and Sirdah models demonstrating notably low specificity (below 54.0%) while maintaining reasonable sensitivity (ranging from 74.2% to 87.8%). This discrepancy raises concerns about the potential for false-positive diagnoses. Conversely, the Kandhro I and Keikhaei models achieved an excessive sensitivity of 100%, yet they were unable to identify negative cases, resulting in 0% specificity completely. Additionally, the Huber-Herklotz model failed to detect any BTT cases, as evidenced by its 0% sensitivity.

**Table 7:** Comparative performance metrics of Basrah Score and conventional scores for IDA vs. BTT discrimination.

**Model**	**Confusion matrix**	**Accuracy**	**Precision**	**Recall (sensitivity)**	**Specificity**	**F1-score**	**AUC**
Basrah Score	[[206 3] [11 210]]	0.967	0.986	0.95	0.986	0.968	0.989
Mentzer	[[24 185] [51 170]]	0.451	0.479	0.769	0.115	0.59	0.442
Srivastava	[[7 202] [56 165]]	0.4	0.45	0.747	0.033	0.561	0.390
Ehsani	[[14 195] [57 164]]	0.414	0.457	0.742	0.067	0.566	0.404
England and Fraser	[[81 128] [7 214]]	0.686	0.626	0.968	0.388	0.76	0.677
Kandhro I	[[0 209] [0 221]]	0.514	0.514	1	0	0.679	0.5
Sirdah	[[36 173] [27 194]]	0.535	0.529	0.878	0.172	0.66	0.525
Keikhaei	[[0 209] [0 221]]	0.514	0.514	1	0	0.679	0.5
Huber-Herklotz	[[187 22] [221 0]]	0.435	0	0	0.895	0	0.447

**Figure 4:**
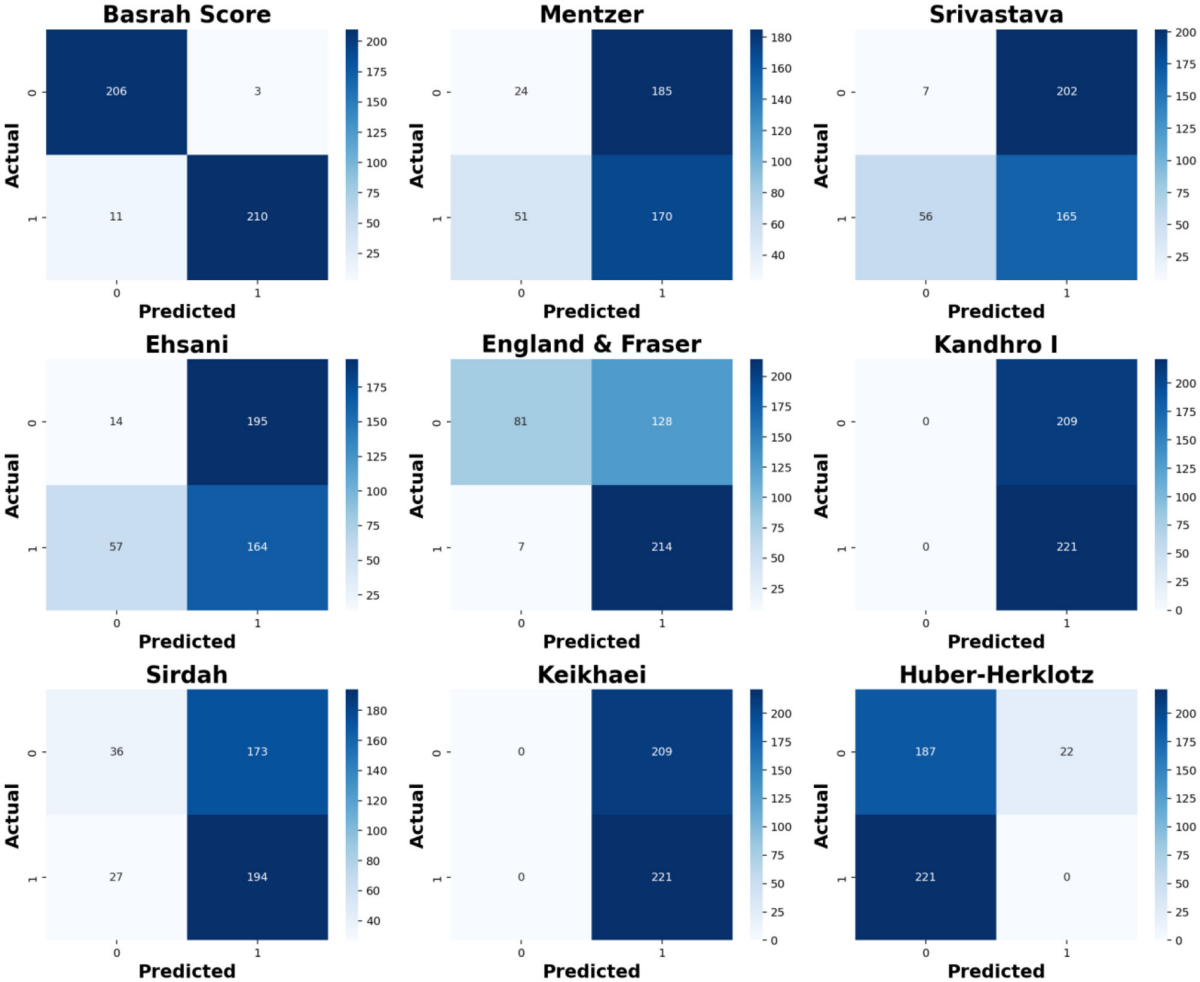
Confusion matrices for different scores.

The following Figure 4 shows, in clear visualization, the comparison illustrated in the confusion matrices, revealing varying performance levels among different models in discriminating IDA and BTT cases. Basrah Score outperformed others, achieving correct classifications for 206 out of 209 IDA cases and 210 out of 221 BTT cases, indicating high accuracy. In contrast, the Ehsani model demonstrated significant weaknesses, misclassifying 57 BTT cases as IDA. Traditional scores such as Sirdah and England & Fraser showed some improvement but remained less effective than the new score. Additionally, the Keikhaei, Kandhro I, and Huber-Herklotz scores exhibited inconsistent performance, failing to classify one of the categories correctly, thereby underscoring the superiority of machine learning-based Basrah Score in this diagnostic task.

**Figure 5:**
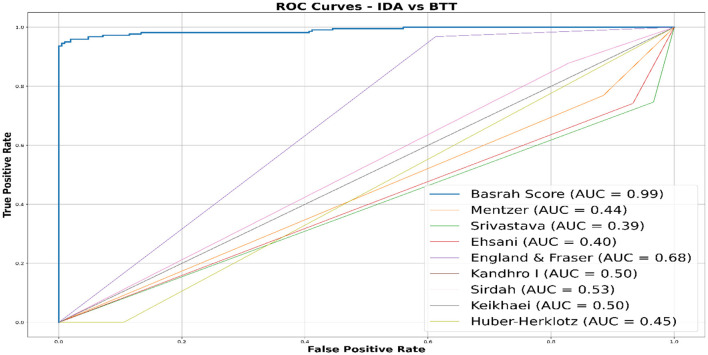
Comparative ROC curves: evaluating model performance in distinguishing IDA and BTT.

Figure 5 illustrates the ROC (Receiver Operating Characteristic) curves for a range of discrimination scores. The figure highlights the superiority of Basrah Score, which boasts a high AUC (~0.99), reflecting its excellent accuracy and robust capability to differentiate between disease cases. In contrast, traditional scores exhibit low AUC values (≤0.680), indicating their limited discriminative power, akin to random guessing. This comparison underscores the significant improvement offered by machine learning Based Score, such as Basrah Score, over traditional scores, thereby enhancing clinical diagnostic accuracy and reducing the likelihood of misdiagnosis.

### 3.4 Check Basrah Score stability

**Figure 6:**
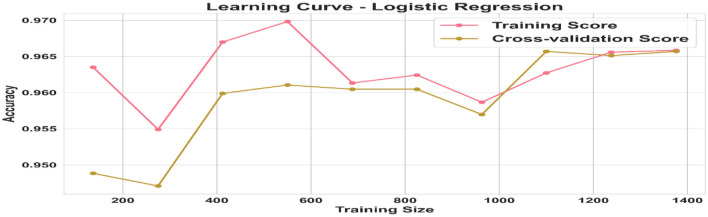
Illustrates the learning curve and stability of Basrah Score with cross-validation compared to its performance without cross-validation.

The learning curve for Basrah Score is illustrated in Figure 6, with training and cross-validation accuracy plotted on the vertical axis against the size of the training dataset on the horizontal axis. Initially, training accuracy is high with a small dataset but tends to decline as the dataset expands, suggesting minimal overfitting. Conversely, cross-validation accuracy begins lower but improves with increased training data, indicating enhanced model generalizability. As the dataset grows larger, both accuracies converge, signaling stabilization of the model. These findings underscore the significance of selecting an appropriate dataset size to reduce overfitting and optimize cross-validation performance, ensuring accurate classification of unseen data.

### 3.5 Results explanation

**Figure 7:**
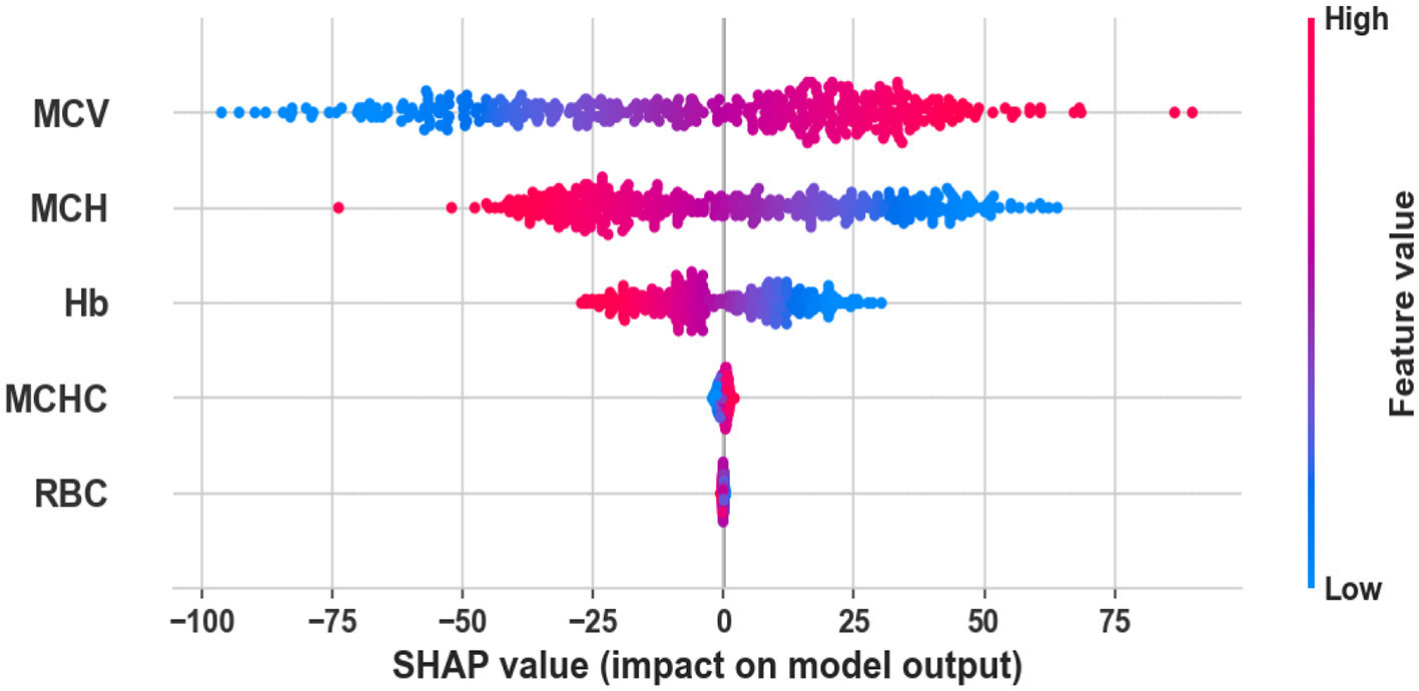
SHAP value analysis: impact of blood parameters on Basrah Score predictions.

The SHAP analysis results (Figure 7) highlight MCV, MCH, MCHC, and Hb as the most informative and discriminative features for distinguishing between IDA and BTT. In contrast, RBC count exhibits significant value overlap between the two conditions, limiting its diagnostic utility when used in isolation. These findings are consistent with established clinical understanding: patients with BTT typically exhibit markedly reduced MCV and MCH values that are disproportionately low relative to their Hb levels, while individuals with IDA present with progressive reductions across both Hb and red cell indices, including MCH, and RBC count.

### 3.6 The impact of a balanced and outlier-removed dataset on performance

This study's results highlight the importance of data pre-processing in improving the performance of machine learning models, especially in medical applications where diagnostic accuracy and reliability are paramount. In the context of using ENLR score to differentiate between IDA and BTT—two clinically similar conditions—it was found that selecting an effective data pre-processing approach significantly enhanced performance metrics. In Table 8, although the original imbalanced dataset exhibited a strong discriminatory capability (AUC = 0.970), the class imbalance led to a lower sensitivity (Recall = 0.869), indicating that some positive cases were missed (False Negatives). Conversely, when the dataset was balanced using the SMOTE technique, there was a modest increase in accuracy (Accuracy = 0.928) and specificity (Specificity = 0.991); however, this approach unexpectedly resulted in a decrease in sensitivity (Recall = 0.856). This reduction may be due to the bias introduced by generating synthetic samples, which can compromise the model's capacity to differentiate between class boundaries accurately.

**Table 8:** The impact of balancing and outlier-removing of data on performance.

**Approach**	**Confusion matrix**	**Accuracy**	**Precision**	**Recall (sensitivity)**	**Specificity**	**F1-score**	**AUC**
Original file without balance	[[218 8] [26 172]]	0.920	0.956	0.869	0.965	0.910	0.970
Balanced with SMOTE	[[229 2] [29 172]]	0.928	0.989	0.856	0.991	0.917	0.982
Balance and remove outliers	[[206 3] [11 210]]	0.967	0.986	0.950	0.986	0.968	0.990

### 3.7 Comparison with another studies

Compared to other studies (Pullakhandam and McRoy, 2024; Shahmirzalou et al., 2024; Al-Najafi et al., 2022) that employed linear and logistic regression, as illustrated in Table 9 below, it is evident that all these studies utilized imbalanced data, which may influence decision bias or lead to overfitting. Furthermore, our analysis demonstrates that this approach outperforms all other methods across various performance metrics, except for achieving comparable accuracy to the proposed model in Pullakhandam and McRoy (2024).

**Table 9:** Comparison with other works.

**Approach**	**Used methods**	**Dataset size**	**Accuracy**	**Precision**	**Recall (sensitivity)**	**Specificity**	**F1-score**	**AUC**
Géron (2017)	LR	Balanced by SMOTE 972 IDA, 19,203 non-IDA	97%	84%	58%	Not explicitly stated	Not explicitly stated	99%
Fawcett (2006)	LR	Imbalance 219 IDA, 73 BTT	93%	Not explicitly stated	97%	72%	Not explicitly stated	Not explicitly stated
Wang et al. (2021)	Binary LR	Imbalance 802 IDA, 578 BTT	85.6 %	Not explicitly stated	85 %	86 %	Not explicitly stated	0.921
Basrah Score	ENLR	Balance 1080 IDA, 1080 BTT	96.7%	98.6%	95%	98.6%	96.8%	99%

## 4 Discussion

This research introduces a comprehensive machine learning framework to distinguish between IDA and beta-thalassemia trait (BTT) and develop a new discrimination score, Basrah Score, effectively overcoming significant shortcomings of traditional discrimination scores. Our results highlight three significant advancements in this hematological diagnostic score. First, the ENLR model exhibited outstanding discriminative ability, achieving an area under the curve (AUC) of 0.990 ± 0.005, which significantly surpassed all conventional indices. This finding supports recent studies that advocate for regularized regression techniques in contexts characterized by high collinearity. Importantly, our model achieved a balanced sensitivity of 95.0% and specificity of 98.6%, representing a substantial improvement over traditional methods, which often displayed unacceptably low specificity or failed to detect either class entirely.

In the context of distinguishing between IDA and the genetic trait of BTT, the predictive performance of the new Basra index was evaluated against traditional discrimination indicators. The findings revealed a 67% improvement in predictive accuracy compared to conventional, static methods, marking a significant advancement in integrating data-driven approaches into clinical decision-making. This progress underscores the growing importance of employing advanced analytical techniques and machine learning in healthcare research, given their ability to uncover complex patterns within clinical data and develop highly accurate and efficient predictive models. Such systems hold particular promise for enhancing diagnostic differentiation in various clinical settings, including pediatric cases, anemia during pregnancy, and chronic diseases.

Furthermore, Basrah Score's decision-making process was validated through SHAP analysis, revealing that mean corpuscular volume (MCV), Mean Corpuscular Hemoglobin (MCH), and hemoglobin (Hb) were the primary discriminators, aligning with established thalassemia biomarkers. The red blood cell (RBC) count had a lesser impact, underscoring its limited diagnostic value when considered alone. Additionally, the automated exclusion of demographic variables such as sex and age allowed for a more focused clinical interpretation of complete blood count (CBC) parameters. Our systematic evaluation of data pre-processing techniques indicated that removing outliers had a more significant effect on model performance than class balancing alone, with the best results achieved through a combination of both methods, leading to the highest accuracy and a balanced F1-score. This finding challenges the prevailing notion that synthetic minority over-sampling technique (SMOTE) consistently enhances minority class detection.

The new scoring equation developed from this study offers a practical tool for clinical laboratories, facilitating the differentiation between IDA and BTT. The equation is expressed as logit(p) = 0.974 + 3.382 × MCV – 5.553 × MCH – 4.228 × Hb, providing a straightforward implementation pathway for enhancing diagnostic accuracy in clinical settings.

The main limitations of this work are the limited number of features (laboratory tests) used in building the model, in addition to the size of the data sample, as expanding the size of the input data contributes to enhancing the model's ability to extract more generalized and accurate decisions due to its exposure to a wider variety of clinical cases. Another limitation is that application is limited to one local dataset as it would be ideal to evaluate the model on multiple datasets from diverse environments to assess its performance and effectiveness in a number of real-world situations.

In light of the current limitations, several future prospects exist for developing this work and enhancing its accuracy and generalizability. The most important direction is to increase the sample size by including data from multiple medical sites, local and global, and classified as populations, including children, expectant mothers, and individuals with long-term illnesses, across different time periods. This will enhance the model's dependability, generalizability and, to validate its stability in different application contexts is also an important step. It is also recommended to include additional attributes in the model, such as advanced tests (such as Ferritin and HbA2), genetic factors, and family history, to increase the predictive power and discrimination accuracy. Furthermore, developing a user interface as a web site that is easy to integrate with medical systems is a practical step toward applying the model in clinical settings. Model interpretation techniques such as SHAP should continue to be emphasized to enhance transparency and medical confidence. In addition, the potential of Deep Learning models and hybrid systems can be explored and compared to the current model, provided that interpretability is maintained. Finally, it is recommended to conduct Prospective Studies and test the integration of the model into Clinical Decision-Support Systems (CDSS) to assess its effectiveness in real-world clinical practice.

This research offers an important pathway to a new generation of medical diagnostic tools that balance the accuracy of artificial intelligence models with the transparency of clinical decision-making processes. The evidence provided indicates that machine learning-based models have considerable potential as clinical decision support aids, given that these models have transparency, interpretability and are perceivable for existing clinical workflows. This allows clinical trust in the decision support tool to develop with greater velocity, therefore accelerating the use and implementation of machine learning-based models in actual clinical practice.

## Data Availability

The original contributions presented in the study are included in the article/supplementary material, further inquiries can be directed to the corresponding author.
